# Mr. James on Inflammation

**Published:** 1832-10-01

**Authors:** 


### Observations on the General Principles, and on the Particular Nature and Treatment of various species of Inflammation. By *J. H. James*, Surgeon to the Devon and Exeter Hospital, and Consulting Surgeon to the Exeter Dispensary. 8vo., pp. 558. London, 1832.

It is now ten years since Mr. James published the first edition of his work on Inflammation, and the interval appears to have been employed in collecting information. The present volume is of magnitude much superior to its predecessor, and we do not doubt that its increase of actual value is in a ratio not very disproportionate to the augmentation of size. We do not propose to analyze this edition, nor even to review it. Its composition is too elementary for such a purpose, and, being a second edition, we should travel over ground familiar to many of our readers. Yet we cannot permit so large an accession to the stock of professional knowledge to pass unnoticed; such a course would be unjust to the author, our readers, and ourselves. We shall select and present an interesting or useful passage here and there, without attempting to connect and unite into the shape of a consistent whole, the heterogeneous fragments.

### State of the Vessels in Inflammation.

The determination of this state would be to a great degree the determination of the long and eager disputes on the proximate cause of inflammation. The prevailing opinion at present appears to be, that the capillary vessels are weakened, and dilated, because, by reason of that weakness, they are unable to resist the *ordinary* force of the heart and large vessels. This has always appeared to us a fallacious theory, although we are willing to allow that it is a specious one. The proper method of forming a theory of inflammation, is, by carefully studying phenomena, and drawing deductions as rigorously precise and germane to the truth as possible. We have not space nor time to enter on this discussion at present, yet we may be permitted to allude to some of the opinions and arguments of Mr. James. This gentleman, like ourselves, doubts the theory to which we have alluded. Let us glance at the appearances presented, under favourable circumstances, to the eye.

Inflammation may be excited by various irritants in parts where its progress may be watched, as in the web of the frog's foot, or the mesentery of hot blooded animals.

"At first, the capillary vessels contracting, the circulation is accelerated towards the point irritated. They then become dilated; after a time the motion in them is lessened, becomes oscillatory, and in some cases is stopped altogether, the globules showing a disposition to coalesce; but it is also stated, on good authority, that while in the centre there is cessation of motion, around this the blood moves more slowly than natural; but still further, *with more than ordinary speed.*" 13.

It is evident, then, that the capillary vessels are dilated, and that there is an unnatural quantity of blood in the part. On this all parties are agreed; but beyond this there is no unanimity nor consent. The question is as to the existence of local action, or the reverse. We do not think that the debility or otherwise should be limited by either party to the capillaries; their object should be to decide on the condition of the *smaller vessels*, including the capillaries; to ascertain whether the circulation through them is accelerated or retarded, augmented in force, or diminished. This seems to us to be the real and practical question. It is idle and unprofitable to discuss the existence of action or inaction in the capillary tubes; we know not strictly what their natural powers are, and consequently we possess no accurate standard of comparison. But it is of importance to ascertain the state of the circulation in the part. If this be decidedly active, and we cannot but imagine that in a fair instance of phlegmonous inflammation it is so, it signifies comparatively little what abstract notions we may entertain respecting the action of the capillaries.

Let us look at the phenomena of phlegmonous inflammation, 1st. *Heat.* It is generally supposed that this is produced by the greater afflux of blood from the interior, because it is known that the blood of the interior is of a higher temperature than that of the surface. And, setting aside the satisfactory experiments that have been made by John Hunter and others, this view of the cause of increased heat, is corroborated by the fact, that the cheek, when suffused with a passing blush, does glow under the temporary accession of blood from the internal parts. But if there were a stasis of blood in the inflamed part, the heat would not be maintained, for the blood being placed under the same circumstances as the surface of the body would lose its temperature, as it does. We may infer then, that not only more blood does arrive at the part, but also that it circulates through it with more than usual rapidity. If this be denied, then we cannot explain the phenomena of increased heat by the state of the local circulation. Throbbing, perceptible and painful, is an accompaniment of heat in inflammation. This is explained by the impulse of the heart being more sensibly felt in the dilated and weakened vessels. It is certainly present when the action of the heart is not manifestly augmented, and those who have suffered from whitlow know that it is not confined to the point immediately inflamed. It usually ceases when suppuration is established; yet we think it would be difficult to prove that the vessels then cease to be weak, if they were weak before, or to be delated, if before dilated by the action of the heart. Mr. James remarks, that if the blood be pressed out of an inflamed part, it will return with great velocity when the pressure is withdrawn. In parts reddened from cold, where there is evident congestion, the contrary is the case.

2. *Redness.* An inflamed part, if formerly white, becomes more or less red, and if previously red, it is rendered redder. This arises, of course, from the greater quantity of red blood which is in it. But we must not stop here. Venous, blood and arterial differ in colour, and, for precisely the same reason, blood circulating slowly is less florid than blood circulating rapidly. In phlegmonous inflammation the colour is vivid, not only because there is much arterial blood in the part, but also because that arterial blood is frequently renewed; in other words, because it is circulated with rapidity. If an incision be made into an inflamed part, all surgeons are aware that not only does more blood escape, but that it escapes with unusual force from vessels of all calibre. It may be said that this is owing to the increased propulsive power of the heart. In inflammation sympathetically affecting the vascular system this is partly true, but in slighter inflammations, where observation can detect no alteration whatever of the general circulation, such a supposition cannot be received. It is difficult to suppose that the weakened vessels, unassisted by increase of force in the heart, would not only bleed more freely, but throw out their blood with more power.

3. *Swelling.* The considerations which present themselves on this phenomenon, do not prove or disprove the theory of inflammation at issue.

4. *Pain.* The pain attending acute inflammation is familiar; it is active, acute, energetic. It is explained by the pressure exercised on the nervous filaments by the enlarged blood-vessels. Probably this is not the whole truth. As the nervous system is the system of relation, that which informs both the animal and the organic life of impressions from without, it seems reasonable to imagine that the external irritant which has occasioned the phenomena of inflammation displayed by the vascular system, has also contributed to excite the sensibility of the nerves. However, this is a subject too extensive and also too subtle for us to enter on. It is evident that the nervous system is intimately linked with the vascular in inflammation. The pain attending the different varieties of inflammation varies in intensity and kind; it differs in acute pleurisy and in rheumatism, in erysipelas and in phlegmon. In certain kinds of inflammation which are called latent, pain is frequently absent. We have seen a dozen, aye, several dozen instances, in a large London Hospital, of death from latent pleuro-pneumonia, where there was little or no pain during life. In acute phlegmonous inflammation not only is the pain pungent, but the ordinary sensibility of the part is excessively increased. Now we know that such an increase argues an augmentation of the powers of the nervous system. But such an augmentation is hardly consistent with debility in the vascular system, and what is applicable to the two systems considered each as a whole, seems fairly applicable to their parts. Thus, latent inflammations usually occur in persons exhausted by typhus, by the discharge from compound fractures, &c. and it may be imagined that it is this exhaustion, shared by the vascular and nervous systems, which renders latent inflammations unaccompanied by the ordinary nervous sensibility, and indomitable by the depletion which generally puts down vascular action. Yet with all these facts before us, we are asked to assent to the opinion that inflammation is in *all* cases essentially the same; that in every instance the smaller vessels are weakened and dilated, the circulation in them retarded.

When we look at the consequences of inflammation and its final cause, we see no better support for the theory we are controverting. Growth we suppose to be an active process, as it is most vigorous in youth and health, most feeble in debility and disease, almost annulled in age. There is a strong analogy between growth and reparation. During the reign of one the other is most powerful. Reparation is essentially effected by inflammation. Growth is not actually inflammation, because there are wanting in it some of the phenomena, and most of the consequences attending that process. But growth, although not inflammation, is a state approaching to it. The vessels in growth become enlarged, the circulation active, the nervous system excitable. The period of growth is the period for phlegmonous inflammation, and, as we said before, for reparative inflammation in its perfection. Here there is a strong analogy between the phenomena displayed by the vascular system in growth and in inflammation. Analogy is not proof, but it is not to be discarded in discussions, and it is always subsidiary to proof. In growth few will pretend to say that the vessels are weakened, for they are then most active, yet in inflammation, analogically comparable to growth, we are told that they are so.

Hitherto we have considered general growth, but the phenomena of partial growth, that of tumours for instance, should not be passed unnoticed. In it the blood vessels become dilated, tortuous, the nervous sensibility occasionally increased. If we admit, and it cannot be denied, that there is general action of the vascular system in general growth, we may well imagine that there is local action of the same system in many instances of partial growth. But it will be found that, by a wise provision, local growth has a still greater connexion with inflammation than general growth. Thus morbid growths have a great tendency to inflame, and to be affected with the consequences of inflammation. Hence nature attempts to destroy them by an extension of the very same process which has produced them.

The original final cause of all inflammation is probably reparation. The good God that created the animal, stamped upon his organization a tendency to actions and a power of originating them, adapted to the repair of a certain amount of injury. But it seems paradoxical to conceive, that what in itself must imply energy and vital power, should be effected by debility of the repairing agents.

The consequences of inflammation are, effusion of the albumen and fibrin of the blood—effusion of pus—effusion of new substances, as of lithate of soda in gout, the ivory deposit in certain cases of diseased joint, the phosphate and carbonate of lime in periostitis—ulceration—granulation and cicatrization—and finally mortification. Some of these processes may argue debility in the smaller vessels, others can hardly do so. Look, for instance, at the inflammation after fracture; see the bone-earth deposited, encased, moulded, and at length fashioned into nearly its natural form and consistence. Here is a process half growth half inflammation; and it does seem to us that the smaller vessels have a power conferred on them which they did not previously possess, rather than that they are deprived of their usual quantum of it. We give this as an apposite example, but the reparative process is in principle the same, whether a bone have been broken or a finger cut, whatever tissue, in short, it is exerted in.

Treatment, philosophically and physiologically considered, is an experiment. The great Bichât looked at it in this light, and in a mood so happy he was prosecuting his inquiry into the Materia Medica, when he fell a victim to his zeal and almost superhuman assiduity. The object of all our theories is the proper and successful application of remedies; the application of remedies not only furnishes a test of the soundness of our theories, but gives us new facts for fresh inductions.

The treatment then of inflammation should teach us something of its nature. It tell us this—that inflammation is not always the same, that acute inflammation is remedied by what empties and relaxes the large vessels and the small—that certain forms of chronic inflammation are best treated by what astringes and gives tone to them. Who does not know that phlegmonous inflammation is best treated by depletion, local, or general, or both; and by the application of warmth and moisture, agents especially calculated to relax. We think this instance sufficient to upset the doctrine which makes inflammation consist in debility of vessels; at least we never heard, nor can our own poor ingenuity devise a satisfactory explanation on such principles. That inflammation is also benefitted by astringents we admit; but it is not that of the most acute kind: and in discussing the proximate cause of inflammation we are taking phlegmonous inflammation as the standard.

We have dwelt enough on this subject. We commenced by alluding to Mr. James's sentiments, and we have been betrayed into an imperfect exposition of our own. We will not sum up with any conclusion, for we honestly confess that the subject yet requires investigation, and that from a man of exact ideas. We think we have said enough to shew that the current doctrine of inflammation is not so consistent nor conclusive as its advocates assert. Our own opinions are of little moment, and we shall not intrude them on our readers; indeed we are too little satisfied with what is known or what we know, to entertain, much more to publish, a definite theory upon the subject. A confession of ignorance is better than a false supposition of knowledge, and the first step towards attaining the latter is to feel assured that we want it.

In the remainder of this short article we shall rather aim at displaying Mr. James's sentiments than our own. As we said before, it is impossible to analyze a work under the circumstances of the present. We may take to this opportunity of suggesting to authors who publish much enlarged editions of their works, to adopt some method of marking the new matter. It is utterly impossible for a reviewer to sit down and compare one edition with the other, in order that he may see what is new and what is old. Dr. Abercrombie and others have judiciously distinguished the additions which they have made, to the advantage of themselves, their critics, and the public.

### Counter-Irritation.

The remarks of Mr. James on this point are sensible and deserving of attention. Two many practitioners use blisters, setons, issues, indiscriminately and at random.

"The subject of metastasis naturally leads to the consideration of another point, namely, the power of artificially substituting one disease for another; for we practically find, that one of the most potent means we possess, consists in establishing a new disease, whereby the other is lessened or removed, by diverting, in fact, the morbid action of the new part.

Two principles seem deserving attention with respect to this point; one is, that the new disease be established at such a distance as not to extend its sphere of irritation to the old one, which will then be augmented by it; the other is, to establish a new disease, approaching as far as may be to the probable nature of the old one; thus, when we wish to remove inflammation which has not proceeded to the effusion of lymph or pus, we should excite simple inflammation, as by a mustard poultice or blister; if it has advanced to secretion from a surface, as in an inflamed joint, a blister will be the appropriate remedy; but if we have to contend with a suppuration or ulcer, then a seton or issue, being of the same kind, will be more likely to cause such a disease as will divert the other.

Nature points out, as has been stated, this mode of proceeding, and I may add, that besides simple inflammation, there are many other morbid phenomena, curative of disease, attributable to the same source; thus, we have hæmorrhoids as well as abscess prope anum; we have hæmorrhages from the nose and premature discharges of the catamenia occurring for the relief of other diseases; and hence we also proceed upon the principle of drawing blood or secretions from a distant part, and this is called revulsion or derivation." 152.

To these remarks we would add, that not only the kind of inflammation and its stage, but the tissue affected, should be taken into consideration in directing the particular counter-irritant to be employed. It is well ascertained that caustic issues are more serviceable in ulceration of the cartilages of articulations than in the strumous disease of their bones, although ulceration be present. Again, when we look to the eye, that repository of illustrations of the effects of disease and of medicine, we observe that blisters are by no means so serviceable in the affections of its mucous membrane, as of the more fibrous textures, the cornea, and sclerotica. This subject has not been hitherto sufficiently investigated; but certainly the better it is understood by the surgeon or physician, the more judicious and successful will his practice be.

### ANGINA EXTERNA.

We notice the short account of this affection, because we have something to add to it. In order that all our readers may know what angina externa is, we will here insert Mr. James's description of it.

"The patient, a person of unhealthy, and generally of full and gross habit, has a swelling deep seated in the side of the neck, generally towards the angle of the jaw, causing a great degree of pain in that side of the head (most probably from its effects on the nerves of the part), and accompanied with much fever. There is loading of the cellular membrane similar to that we observe in E. phlegmonodes, but limited, firmer and more prominent. This takes place to a great degree, and the result is, that the patient is scarcely able to swallow even fluids; breathes with great difficulty, and cannot sleep from the impending suffocation. The inflammation extends itself internally to the mucous membrane of the fauces and uvula, and thence often to the bronchial membrane; in extreme cases also there is great œdema of the face from the pressure on the absorbents.

The skin, which, if we see the case from the beginning, is then apparently unaffected, soon adheres and inflames, and thickens as it inflames, but does not point, or for a long time show any symptom of pointing, or giving way by slough or ulceration; meanwhile extensive sloughs and noisome pus form underneath, and do great mischief. These abscesses may extend into the cellular membrane, within the upper part of the thorax; and I have seen them form on both sides of the throat.

There is great danger of their destroying life, not only from the direct influence of such injurious matters when confined, but by suffocation, if proper relief is not afforded; and although, if left to nature, they will ultimately burst on the surface, or into the throat, the patient may not live for this to take place.

There can be no question that a free incision here is fully as important as in any case of carbuncular inflammation; and although, from the nature of the parts, the surgeon would be unwilling to do this, if the necessity were not paramount, yet on his conduct in this respect, the life of the patient will mainly depend: a person accustomed to these collections of foul matter beneath thickened integuments, will detect its existence by the peculiar feel of a doughy yielding matter under the tense elastic skin.

The only author who had particularly mentioned this form of inflammation that I was aware of, when this work was first published, was Dr. Kirkland; his description is very brief; he gives one case. There have since been others related which I cannot doubt are of the same nature, although by some they are considered as suppuration of the parotids, which I am apt to believe is very uncommon." 339.

In the first place, we object altogether to the name of angina. The old fashion of giving particular and various designations to the same disease in different parts, though in the same tissue, of the body, has been very mischievous. Men have imagined that such multifarious denominations implied diseases as multifarious, and that cellular inflammation, in one spot, was dissimilar to cellular inflammation in another, because it had received peculiar designations. We object, then, to the term angina, because with angina it has little in common. The affection in question is essentially and really a diffuse inflammation of the deep cellular tissue of the throat. We do not speak from speculation, but from observation; we have seen such cases, and have seen them sadly mismanaged. We remember one very severe instance of this affection. The patient was a gentleman's servant; one side of his face was greatly swollen, as was the front, one side of the neck, and even the lateral and upper part of the thorax. The colour of the integument was dusky red, without the defined border of erysipelas. The swelling was of a boggy character, with distinct fluctuation in one part. The complaint had been trifled with for some days, and the constitutional symptoms had assumed a grave, indeed alarming aspect. Free and sufficiently-deep openings were made, pus, stinking gas, and shreds of sloughy cellular membrane, resembling wetted tow, being discharged or exposed. In the face the mischief was subcutaneous, but extended into the adipose cellular tissue of the cheek, and under the masseter muscle. In the neck the cellular texture under the fascia, and beneath the sterno-mastoid muscle, was the seat of suppuration, &c. Ultimately the patient recovered, but he required much support, and the muscles were laid bare extensively by suppuration. The lower jaw was also denuded; but granulations formed on it, and no exfoliation occurred. We lately saw another case, less extensive because more promptly attended to. In this, the matter passed under the sterno-mastoid muscle; and in order to evacuate, it was necessary to make openings and counter-openings through the fascia. The patient did very well.

We say, then, that this affection is essentially inflammation of the subfascial, inter-muscular, and, secondarily, of the subcutaneous cellular tissue. If moderate, lymph is deposited, and a circumscribed abscess results—if more rapid, or occurring in a bad habit of body, it is not so circumscribed, and extends in various directions. It may pass upwards to the side of the face, as in the instance we have mentioned—or under the angle of the jaw to the pharynx—or beneath the laryngeal muscles, towards the sides of the larynx and œsophagus—downwards to the thorax, where it may be stopped by the deep cervical fascia, or travelling along the exterior of the œsophagus and trachea, it may extend down the posterior mediastinum. All these are not possibilities merely, calculated from a glance at the dead body; they are things which have happened, and will happen again.

As the disease invades the deep cellular membrane, if it does not begin in it, the usual characters of inflammation of that texture will be present. There is more or less swelling, of doughy character, with superficial puffiness or œdema in the first instance; at this time the colour of the integuments is little altered. As the disease extends, the swelling becomes more apparent, still continuing hard or boggy rather than elastic, the subcutaneous cellular membrane is implicated, and there is more œdema or even suppuration in it, and the skin assumes a dull inflammatory blush, without the same disposition to vesication, and to a defined and spreading border, observed in erysipelas. At a still more advanced period, the extent of the parts occupied is considerable, distinct collections of matter form, and putrid gas and slough of the cellular tissue are intermixed, communicating, on pressure, a sort of emphysematous crackling beneath the skin.

The local symptoms will depend on the local mischief—its extent, and the parts interfered with; if the neighbourhood of the pharynx is implicated, there will be difficulty of swallowing; if the larynx, dyspnœa and symptoms of laryngitis; and so on. The difficulty of swallowing and dyspnœa are generally present in a greater or less degree. The constitutional symptoms are moderately inflammatory in the first instance—invariably typhoid subsequently.

The treatment is simple, but it must be bold. If the practitioner be called early, large and repeated leeching and antiphlogistic treatment will usually be indicated; bleeding is seldom necessary, or to be recommended. But as soon as induration and bogginess are established, there must be no more delay in resorting to sufficient incisions; we say sufficient, for unless they be carried through the fascia, they are comparatively useless. As soon as adequate incisions have been made, the excitement commonly gives way to depression, and judicious support is demanded. We have thrown together these few observations, because we think Mr. James's account of this affection incomplete, whilst it is indispensably necessary to the surgeon to be accurately acquainted with its characters and treatment.

### CARBUNCULAR INFLAMMATION ON THE FINGERS.

"There is a kind of carbuncular inflammation which occurs on the fingers, not true paronychia, for it is unconnected with the nail, and equally so with the thecæ. It occurs often on the back of the first or second phalanx: it is dusky, excessively painful, and throws up a livid pustule or phlyctena. It may often be imputed to some morbid poison, but I have seen it occur where there was no reason to suppose this. It happens to persons of a bilious temperament and spare constitution. A very foul, sloughy ulcer ensues after the pustule is broken, and a core is often discharged.

Sometimes an incision gives great relief, but I have also seen it aggravate the pain and inflammation. Poultices with opium, entire rest, purgatives, and opium to relieve pain, are necessary; and to the ulcer when formed, sometimes escharotic, at others sedative applications agree best. I may also mention the arsenical lotion.

These seem to bear the same relation to carbuncle, which whitlows in the last phalanx do to boils." 345.

There is another kind of affection of the fingers which we have seen on one or two occasions. It resembles what has been termed the subcutaneous tubercle, the produce of cachexia. At first a small tubercle appears to form under the skin, usually on the backs of the phalanges; the skin becomes blueish ; matter is secreted and the skin ulcerates, and a small circular, whitish, indolent-looking sore, capable of holding a small pea, is the consequence. Many of these sores exist at the same time, one appearing as another is healing up. The circulation is usually bad, the hands and feet cold and purplish, the pulse weak, the whole system languid. What the disease is owing to is difficult to determine. It will exist for two or three months, and then there will be an interval of variable duration before it reappears, to run nearly the same course as before. We have found the decoctum sarsæ comp. with the volatile alkali, the most efficient remedy.

### Inflammations from Poisons.

Poisons, it is well known, are derived from the animal, vegetable, and mineral kingdoms. The former differ from both the latter in being followed by secondary symptoms, and it is clear, from the differences exhibited by those secondary consequences, that the animal poison is not a mere irritant to this or that tissue, but exerts a specific influence. We shall not say more of morbid poisons, than that they form a study of themselves. Man furnishes morbid poisons for man, and some can be communicated independently of inoculation or actual contact. But the morbid poisons fabricated by other animals can only, so far as we know, be communicated by contact, or, in the majority of instances, by direct inoculation. The most formidable animal poisons of this description are those of the snake and of rabid animals. On the latter we need not descant. The bite of the viper being that, among the former, which is observed most frequently in this country, we are tempted to extract Mr. James's brief exposé of the symptoms which follow it, and of the remedial means which have been recommended and employed for it.

"Sudden and great swelling takes place in the part (probably a hand or foot), in the limb, and very speedily in the adjacent part of the trunk. The inflammation appears to affect all the tissues, and very notably the cellular membrane; the skin also is much implicated, the cuticle separates in phlyctenæ, and a remarkable tendency to ecchymosis exists, even to a great distance, in that portion of the body which is affected continuously by the poison, probably from the tone of the vessels being much broken down. Nothing can be more formidable in appearance, and sometimes in reality, than this inflammation, although when produced by the viper it does not often prove fatal.* Not unfrequently, however, some portions of the integuments fall into gangrene, especially in the neighbourhood of the bitten part; but in all cases, resolution takes place through a great portion, if the person survives.

The affection of the general system so speedily follows the bite as to give great reason for the belief that it does not proceed so much from sympathy with the local disease as from the absorption of the poison. Great depression, tendency to syncope, vertigo, sickness and vomiting of bilious matter, cold sweats,

* "The danger will depend, *cæt. par.*, upon the vigour of the poisonous animal, and the relative feebleness of the recipient."

small pulse, and occasionally pain about the navel, are the result, and may increase to a fatal termination, which often occurs in a very short time from the bites of the more venomous serpents; but from that of the viper recovery commonly takes place, and the symptoms begin to subside sooner than might be expected from their severity and the extent of the local disease: they often cease to be formidable within two or three days.

The treatment in these cases consists, 1st. In preventing the communication of the poison to the system. 2d. In moderating its local effects by local applications. 3d. In general remedies.

The first object may be attempted either by the immediate excision of the bitten part, by sucking out the virus by the mouth or cupping-glasses, or by its destruction in the wound by caustic; for which purpose M. Boyer, in his Traité des Maladies Chirurgicales, has recommended the introduction of some liquid caustic into the orifices so as to convert them into sloughs. He employs the muriate of antimony, sulphuric or nitric acid, inserted into the wounds, on wood properly shaped.

This object may be further attempted by arresting the progress of the virus after it has been absorbed, by the application of bandage or ligature, and it is not deemed necessary for this purpose to intercept the circulation altogether. In cases where ligature is used, a portion only of the virus seems to operate on the nervous system, and its effects may then be successfully combated. Of this mode of treatment, we have an interesting account by Dr. Butter, in the second volume of the Calcutta Transactions.* The only objection which suggests itself is the increased chance of mortification in the part: it however appears probable that Nature has the power of conquering those poisons which do not destroy the individual too soon, and that, perhaps, in the part as well as in the system.

The second object, that of overcoming the local effects by local remedies, is attempted by a practice which has resulted from observation. Certain medicines, especially ammonia combined with olive oil, and employed with friction, appear highly serviceable: the ung. hydrarg. is also an application of considerable efficacy. Indigo and many other applications are recommended.

The third purpose is accomplished by supporting the powers and stimulating the nervous system; for this end, ammonia, eau de luce, brandy, mulled wine, laudanum, warmth, frictions, &c. should be employed. Dr. Butter also walks his patients about, as in cases where an overdose of laudanum has been taken, until the patient rallies, and then removes the ligature.

Another plan consists in giving medicines which have some specific quality. The serpentaria has been used with this view; but it probably deserves to be regarded only in the light of a useful stimulus; the guaco is much spoken of, and arsenic has also been freely exhibited, and, it should appear, with much success: from the accounts we have received it is well deserving a trial." 259.

We have an observation to offer respecting the application of escharotics to poisoned wounds. The only experience which we have had of them has been in wounds received in dissection, and that experience makes us decidedly averse to their use under those circumstances. We have seen a most troublesome, ugly sore result from the application of the nitrate of silver to a dissection-wound, and unirritating treatment has always seemed to us the best. Yet here we have reason to believe that an animal poison is received, and, from analogy, we should imagine that the same results would follow in the case of a viper or snake-bite. Analogy, however, is not proof, and the matter must be decided by the balance of evidence. If

---

* P. 221-2.

testimony goes for any thing, arsenic should be the most potent remedy in snake-bites; its reputed effects, after wounds inflicted by the serpent of St. Lucia, are well known to professional readers.

In the stings of gnats, hornets, &c. Mr. James has seen great relief afforded by gently rubbing the part with ung. hyd. and applying extensively a lotion composed of liq. ammon. subcarb. ʒij. aquæ, ℥vj. to the surrounding parts.

Vegetable poisons are seldom very injurious, unless when introduced into the cellular tissue, or applied to parts deprived of cuticle. Even here they act for the most part as mere irritants. Thus port wine and water, injected into the cellular tissue of the scrotum, will kill it; but port wine and water is certainly not a poison under ordinary circumstances. The reason why it kills the cellular texture, is because this possesses a low degree of vitality, and it is destroyed by the injection, which scarcely produces inflammation enough in the tunica vaginalis. Mr. James remarks, that neither urine nor wine will always occasion sphacelus of the cellular membrane, if they do not continue long in contact with it, and, therefore, that the earlier incisions are made to evacuate the fluid the better. This is most true and most important; and we would repeat most emphatically, the earlier and bolder the better.

Mineral poisons act variously, according to the chemical affinity of the agent with the living tissue. Nitric acid and the caustic alkali produce very different sloughs; the former destroys what it comes into contact with, the latter combines with it chemically, and is, consequently, a penetrating caustic. Thus, an issue made with the former is usually of determinate depth—with the latter, it may pass much more deeply than the inexperienced operator would imagine. The tartarized antimony appears to us to act on a particular texture, and it is well known to produce occasionally a sort of secondary eruption of pustules. The texture on which it more immediately acts seems to be the glandular system of the hairs. If a pustule, produced by tartar-emetic, be carefully examined, it will invariably be found to contain in its origin a hair in its centre; such, at least, has ever been the case when we have had the curiosity to inspect it. Arsenic exerts something like a specific operation, producing, when introduced into a wound, inflammation of the mucous membrane of the stomach.

Our limited space compels us to close Mr. James's volume. We recommend it warmly to the attention of students, both in practice and out of it. There is much information to be derived from it. It is not without faults, but they are infinitely overbalanced by its merits; and again we repeat our hearty commendation, and recommendation to our junior brethren to peruse it. It is a pleasant thing to see our provincial surgeons exerting themselves, to prove that knowledge is every where diffused among the ranks of our intelligent profession.

